# Nonconvulsive Status Epilepticus Resembling Clinical Absence with Atypical EEG Pattern

**DOI:** 10.1155/2017/6987821

**Published:** 2017-01-19

**Authors:** Channaiah Srikanth Mysore, Najib Murr, Rana Zabad, John Bertoni

**Affiliations:** ^1^Department of Neurological Sciences, University of Nebraska Medical Center, 988440 Nebraska Medical Center, Omaha, NE 68198-8440, USA; ^2^Department of Neurology, Southern Illinois University School of Medicine, 751 N Rutledge Street, Springfield, IL 62794, USA

## Abstract

*Objective*. We are reporting two cases: a patient with steroid responsive encephalopathy associated with autoimmune thyroiditis (SREAT) and another patient with secondary progressive multiple sclerosis (SPMS), both presenting with altered mental status (AMS) and later diagnosed with nonconvulsive atypical absence status epilepticus (AS), with atypical EEG changes.* Methods*. A report of two cases.* Results*. A patient with history of SREAT and the other with SPMS had multiple admissions due to AMS. For both, EEG revealed the presence of a high voltage generalized sharply contoured theta activity. A diagnosis of NCSE with clinical features of AS was made based on both clinical and EEG features. There was significant clinical and electrographic improvement with administration of levetiracetam for both patients in addition to sodium valproate and Solumedrol for the SREAT patient. Both patients continued to be seizure free on follow-up few months later.* Conclusions*. This is a report of two cases of atypical AS, with atypical EEG, in patients with different neurological conditions. Prompt clinical and EEG recovery occurred following appropriate medical treatment. We think that this condition might be underreported and could significantly benefit from prompt treatment when appropriately diagnosed.

## 1. Introduction

Nonconvulsive status epilepticus (NCSE) is defined as a change in behavior and/or mental processes from baseline associated with continuous epileptiform EEG discharges. The electrographic NCSE in the obtunded patient (also referred to as nontonic-clonic status epilepticus (SE) or subtle SE) consists of at least 30 minutes of marked obtundation or coma, without clonic activity, cyclic changes in behavioral level or level of consciousness, and continuous electrographic seizure activity on EEG [1].

While convulsive status epilepticus (CSE) is associated with significant morbidity or mortality, morbidity in NCSE largely depends on the underlying etiology and circumstances [2].

Absence status epilepticus (AS) shares clinical features with NCSE. Typical ASE occurs in the setting of idiopathic generalized epilepsy (IGE) and should be differentiated from atypical de novo AS which occurs in patients without history of epilepsy yet precipitated by several epileptogenic factors [3]. AS can present with atypical clinical and electrographic features that could delay the diagnosis and treatment of this condition.

In this manuscript, we report two adults who presented with confusion and other neurological signs, found to have NCSE clinically resembling AS with atypical EEG findings, who were successfully treated with antiepileptic drugs (AED).

## 2. Case Reports

### 2.1. Case 1

A 29-year-old man with a three-year history of steroid responsive encephalopathy associated with autoimmune thyroiditis (SREAT) and partial epilepsy, on levetiracetam 1000 mg twice daily, was evaluated for confusion, lightheadedness, and nausea. He had a seizure a few weeks earlier attributed to noncompliance to levetiracetam which was restarted and subsequently increased to 1000 mg twice daily. His medical history is significant for generalized CSE a few years earlier in the setting of SREAT with high levels of thyroid antibodies. This was successfully treated with a tapered schedule of prednisone for six months in addition to levetiracetam that was discontinued without medical advice.

Neurological exam showed a nonfatigable multidirectional nystagmus, short-term memory deficit and a brain MRI demonstrated a Chiari-1 malformation. He was discharged home but was admitted the next day for worsening mental status and intermittent jaw jerking. Neurological exam revealed altered short-term memory, action tremor, and myoclonus.

**Figure 1:**
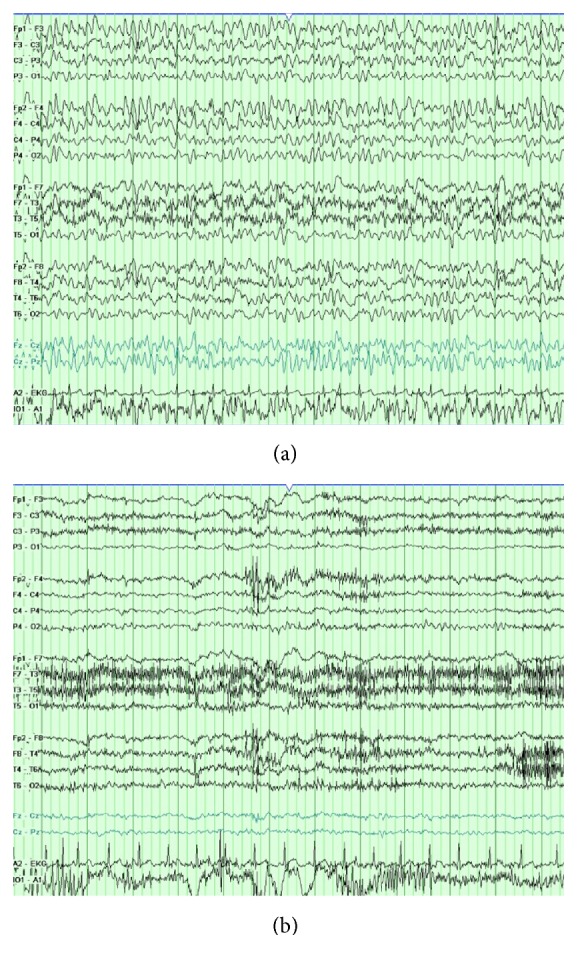
(a) 12 seconds of abnormal EEG showing high voltage generalized sharply contoured theta activity. Voltage scale: 7 microvolts/mm. (b) Improvement following medical treatment.

During this hospitalization, the abnormal laboratory tests included a TSH of 5.32 micro-IU/mL, thyroid microsomal antibody of 2935.1 IU/mL (normal 0–9), and thyroglobulin antibody 75.1 IU/mL (normal 0–20). The EEG performed on day 2 demonstrated high voltage generalized sharply contoured theta activity (Figure 1(a)). Levetiracetam was increased progressively to 2000 mg twice daily but there was no significant clinical or EEG improvement. On day 4, he was started on Solumedrol 1000 mg IV daily and sodium valproate (VPA). By day 5 and after two doses of Solumedrol, his mental status improved dramatically and his EEG normalized (Figure 1(b)). He was discharged home on levetiracetam and VPA. He reported no seizures and had normal thyroid studies five months later upon follow-up. He continued to have persistent short-term memory deficit.

### 2.2. Case 2

A 42-year-old woman with history of secondary progressive multiple sclerosis (SPMS) with spasticity on baclofen, chronic low back pain on pregabalin, chronic depression, and history of seizures treated with phenobarbital up to 5 years of age with no reported seizure recurrence was admitted for word finding difficulty and intermittent confusion. She has been having paroxysmal episodes of altered mental status (AMS) requiring multiple hospitalization in the past year with no clearly identified etiology. The last hospitalization was two weeks earlier following unintentional diphenhydramine overdose. An EEG performed three years earlier was reported to be normal.

**Figure 2:**
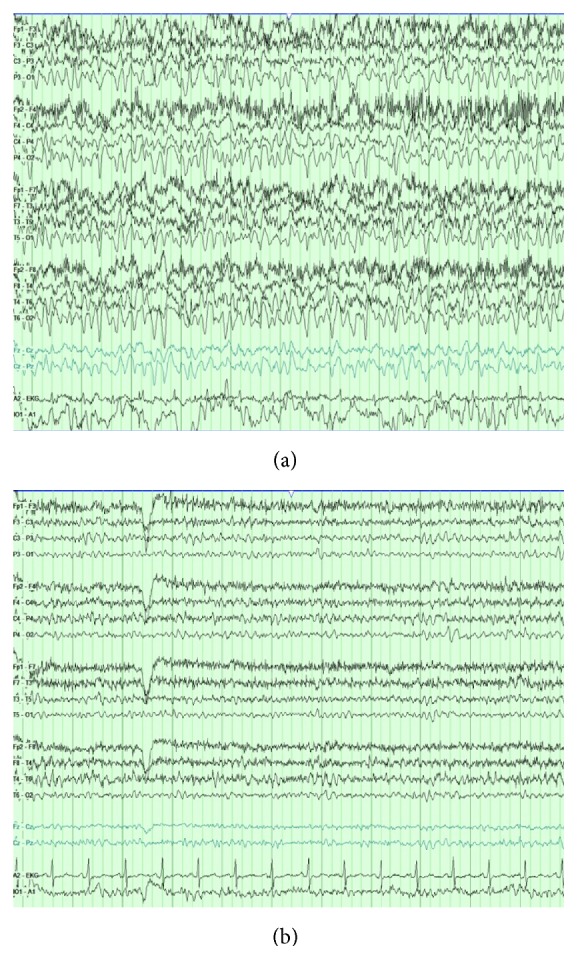
(a) 11 seconds of abnormal EEG showing high voltage generalized sharply contoured theta activity. Voltage scale: 7 microvolts/mm. (b) Improvement following medical treatment.

On the day of admission, pregabalin was stopped and the patient was diagnosed with urinary tract infection, which was treated with sulfamethoxazole/trimethoprim for 3 days. Neurology was consulted for evaluation of AMS. On exam she was oriented only to person and had word finding difficulty. Brain CT scan was unremarkable and EEG showed high voltage generalized sharply contoured theta activity (Figure 2(a)). Levetiracetam 1000 mg was administered, followed by 500 mg twice daily for presumed NCSE. Complete clinical and electrographic recovery occurred a few hours after starting AED therapy (normal EEG in Figure 2(b)). She was discharged home the next day on levetiracetam 500 mg twice daily and her home medication pregabalin 100 mg twice daily was restarted. She reported no seizures upon follow-up 3 months later.

## 3. Discussion

These two cases share electrographic and clinical characteristics with AS and complex-partial status epilepticus (CPSE) and the differentiation between both types can be tricky. The literature on the clinical and electrographic features of absence seizures in adults is scarce. The emphasis in the literature has been on the pediatric population with typical ictal EEG showing 2.5–4 Hz generalized spike-and-wave discharges in typical absence (occurring in the setting of IGE) and slower frequencies in atypical generalized absence seizures (occurring in patients with cryptogenic or symptomatic generalized epilepsy) [3]. AS in adults is infrequently reported in the ambulatory setting; however, there is growing awareness of nonconvulsive seizures as an underdiagnosed cause of AMS in the intensive care units and medical floors [4]. The ILAE proposed four axes for the diagnosis of SE, the first being semiology. The lack of prominent motor symptoms defined NCSE. Further subclassification of NCSE depends on the three other axes consisting of etiology, EEG findings, and age. A symptomatic NCSE is caused by a known disorder (likely SREAT in case-1 and maybe UTI precipitating AS in case-2). As regards to the ictal EEG features, it was clearly stated that the SE ictal patterns are all nonspecific since with longer duration of SE, nonepileptiform patterns could become predominant and therefore a detailed description of the suspected ictal patterns in addition to response to medical treatment is very helpful in making a diagnosis [5].

Generalized nonspecific ictal EEG changes were reported with three cases of NCSE of limbic origin [6]; however, limbic semiology was not displayed by any of our patients.

In the case of AS, the duration of symptoms prior to making the diagnosis may range from minutes to weeks [7]. In fact our second patient had fluctuating mental status, for almost a year prior to the diagnosis.

In most cases of CPSE, evolution of clinical symptoms is gradual and reports suggest a temporal origin.

AS with atypical features can be seen in patients with idiopathic generalized epilepsy (IGE) and is sometimes triggered by initiation or dosage increase of AED, such as carbamazepine, gabapentin, phenytoin, or vigabatrin [8]. In both of our patients, there was no exposure to those AEDs and IGE was not diagnosed in either case.

The EEG may not always help distinguishing between different types of NCSE. EEG features in AS can include 3 Hz generalized spike-wave discharges, 10–15 Hz generalized rhythmic discharges, or other forms of rhythmic epileptiform activity [9, 10]. Generalized and focal EEG abnormalities were reported with CPSE as mentioned earlier in the discussion.

Typical ASE is usually treated successfully by intravenous administration of diazepam or lorazepam. If seizures continue for more than 10 min, repeated doses can be administered. Eventually, administration of other longer acting AEDs such as VPA, phenobarbital, or other AEDs should be considered [1, 11].

Owing to the favorable clinical outcome in patients with NCSE, caution should be taken with further treatment with intravenous anesthetics. Aggressive pharmacological treatment may lead to greater morbidity and mortality than nonconvulsive seizure activity itself [12].

It is worth mentioning that myoclonic SE has also been reported with pregabalin treatment [13], which is not what our second patient had.

Back to our patients, it is likely that the addition of steroid therapy had contributed to AS resolution in case-1 given the underlying SREAT “flare-up,” and the coexistence of SPMS and prior diagnosis of epilepsy in case-2 is likely to be coincidental.

In conclusion, in certain cases of NCSE, a high index of clinical suspicion is warranted for appropriate diagnosis and treatment despite the presence of nonspecific EEG patterns. Normalization of both clinical and electrographic features following appropriate medical treatment can be diagnostic. Finally, we think that NCSE clinically resembling ASE could be an underreported and undertreated condition.
